# Protein tyrosine phosphatase 1B(PTP1B) promotes melanoma cells progression through Src activation

**DOI:** 10.1080/21655979.2021.1988376

**Published:** 2021-10-25

**Authors:** Qiang Wang, Yuyan Pan, Liping Zhao, Fazhi Qi, Jiaqi Liu

**Affiliations:** a Department of Plastic Surgery, Zhongshan Hospital, Fudan University Shanghai, China; b Department of Plastic Surgery, the First Affiliated Hospital of Ustc, Division of Life Sciences and Medicine, University of Science and Technology of China, Hefei, Anhui, P.R. China

**Keywords:** Melanoma, ptp1b, src, metastasis

## Abstract

Previous studies have demonstrated that protein tyrosine phosphatase 1B (PTP1B) can promote tumor progression in breast cancer, colon cancer and prostate cancer. Additionally, PTP1B also acts as a tumor suppressor in esophageal cancer and lymphoma. These findings suggest that PTP1B functions as a double-faceted molecule in tumors. However, the role of PTP1B in malignant melanoma (MM) is still unknown. PTP1B expression in normal and melanoma tissues was evaluated by GEO analysis and immunohistochemistry. The effects of PTP1B on cell migration and invasion were evaluated in melanoma cells with up – and downregulated PTP1B expression. In this study, we initially demonstrated that the expression of PTP1B in malignant melanoma tissue is significantly higher than its expression in benign nevus tissue and indicated poor survival of malignant melanoma patients. In vitro studies have demonstrated that inhibition of PTP1B suppresses and overexpression of PTP1B promotes migration and invasion of melanoma cells. Moreover, we found that PTP1B could interact with Src via coimmunoprecipitation and dephosphorylation of the Src at Tyr530 site. Collectively, our study revealed that PTP1B can promote melanoma cell metastasis by interacting with Src and provides a theoretical basis for future applications of PTP1B inhibitors in the treatment of malignant melanoma.

## 1. Introduction

Cutaneous malignant melanoma is one of the most lethal cancers in the world, and its incidence has increased in recent years [1,2]. Localized melanoma can be treated quite successfully with surgery but will metastasize rapidly if not caught early [3]. Although targeted therapy agents such as BRAF and MEK kinase inhibitors, as well as immune checkpoint inhibitors, have been widely used in the treatment of malignant melanoma, many patients with metastatic melanoma cannot achieve tumor remission [4,5]. Thus, it is urgent to explore potential therapeutic targets, elucidate the biological mechanism underlying tumorigenesis and cancer progression, and develop novel therapeutic strategies against malignant melanoma.

Protein tyrosine phosphatase 1B (encoded by PTPN1) is recognized as a potential therapeutic target for the treatment of diabetes, obesity and cancer [6,7]. Numerous studies have reported that PTP1B-deficient mice are hypersensitive to insulin and resistant to obesity induced by a high-fat diet [8]. In addition to its role in regulating insulin, PTP1B has a dual role in cancer development. PTP1B expression was increased in prostate [9], breast [10], ovarian [11], lung [12], colon [13], gastric [14] and pancreatic [15] cancers. In colon cancer, PTP1B has been shown to activate Src by dephosphorylating its negative regulatory residue [13]. PTP1B has also been shown to be a positive mediator of ErbB2-induced signals that trigger breast tumorigenesis and to be required for ErbB2 transformation in breast epithelial cells [16]. In contrast, PTP1B acts as a tumor suppressor in hematopoietic malignancies, such as classical Hodgkin’s lymphoma and B-cell–like diffuse large-cell lymphomas [17,18]. However, the association between PTP1B and MM has never been described.

Src is one of the members of the Src kinase family and plays an important role in cell proliferation, differentiation, movement and localization [19]. Its three main domains are the Src homology domains SH2 and SH3 and catalytic domain, which contains an active kinase. Both SH2 and SH3 are required for protein-protein interactions. A large number of studies have found that the human Src gene product c-Src is overexpressed and highly activated in a variety of human tumor cells, and Src activation promotes tumor progression [20]. Src is activated by protein–protein interactions or phosphorylation events. Tyr419 and Tyr530 are the two main phosphorylation sites in Src [21]. Tyr419 can be internally phosphorylated in the presence of a mitotic signal that removes the activation ring from the catalytic pocket. This allows phosphorylated Src to activate downstream targets that promote cell proliferation [22]. Phosphorylation of Tyr530 by other proteins negatively regulates Src and inactivates its kinase activity by the blocking catalytic pocket [23]. However, the biological functions of PTP1B and Src in MM are unknown.

In this study, we identified PTP1B as a tumor promoter in which PTP1B expression increases with tumor progression. We detected the expression of PTP1B in human melanoma tissues and evaluated its function in melanoma metastasis in vivo and in vitro. Moreover, human phospho-kinase array and co-IP analysis revealed a critical role for PTP1B in melanoma metastasis through dephosphorylating Src.

In the present study, we hypothesized that PTP1B is involved in the metastasis of MM by activating Src. To prove this hypothesis, we evaluated the expression of PTP1B in human melanoma tissues and detected its effects on melanoma cell migration and invasion in vivo and in vitro. Additionally, the relationship between PTP1B and Src in MM was investigated.

## 2. Methods

### 2.1 Cell culture and transfection

The human melanoma cell lines MV3 and A2058 used in this study were obtained from ATCC. MV3 cells were cultured in RPMI 1640, and A2058 cells were cultured in DMEM; both media were supplemented with 10% FBS, 100 IU/mL penicillin, and 100 mg/mL streptomycin. Cells were maintained at 37°C with 5% CO2. PTP1B-targeted shRNA (shPTP1B, 5′ – GGAAGAGACCCAGGAGGATAA-3′) was synthesized to knock down endogenous PTP1B expression in melanoma cells, and a nonhuman homologous shRNA sequence was used as a negative control (shCon, 5′-TTCTCCGAACGTGTCACGT-3′). For the generation of lentivirus, 293 T cells were transfected with optimized packaging plasmid (pAXA2, pVSV-G) along with shPTP1B or shCon vector by Lipofectamine (Invitrogen). After 72 h, virus-containing medium was collected and used for infection of target cells in the presence of polybrene. Then, the stably infected cells were selected by puromycin (5 μg/mL).

### 2.2 Patient samples

The tissue microarray was obtained from Xi’an Alenabio.com (China), and detailed information on this microarray has been previously described [24].

### 2.3 Immunohistochemistry

The paraffin-embedded sections were deparaffinized, rehydrated and then exposed to the primary antibody (PTP1B,1:100 dilution) at 4°C overnight. After they were washed three times with phosphate-buffered saline (PBS), the sections were incubated with the secondary antibody at room temperature for 30 min, followed by treatment with a 3-amino-9-ethylcarbozole (AEC) solution for 1 min. Then, the sections were stained with hematoxylin to identify morphological changes. The PTP1B staining intensity was independently evaluated and scored by two pathologists. The IHC score was calculated as previously described [25].

### 2.4 Cell proliferation assay

Cell proliferation was detected with a cell counting kit-8 assay (CCK-8, Dojindo). The cells were seeded in a 96-well plate in RPMI 1640 supplemented with 10% FBS. The absorbance was detected at 450 nm on a TECAN microplate reader (Mechelen, Belgium).

### 2.5 Migration and invasion assay

Cell migration was detected with the wound healing assay or Transwell assay as previously described [26]. Cell invasion was detected using FalconTM Cell Culture Inserts (BD353097, BD company, USA, New Jersey) according to the manufacturer’s instructions. First, 100 μl of Matrigel (BD Biosciences, USA) (5 mg/ml) diluted with 500 μl of DMEM was added to the upper chamber of the insert and incubated for 60 min at room temperature. Cells suspended in DMEM at a density of 1 × 106 cells/ml were then added to the upper chamber (100 μl/each), while 500 μl of medium was added to the lower chamber. After incubation at 37°C for 24 h, the cells on the membrane surface of the upper chamber were carefully removed, and cells on the bottom surface were fixed in 10% formaldehyde for 15 min, stained with 5% crystal violet for 30 min and observed under a microscope. The numbers of infiltrating cells were counted and photographed from five randomly selected fields. All experiments were performed in duplicate and repeated three times.

### 2.6 Co-immunoprecipitation

Whole-cell lysates were precleared and incubated with mouse anti-HA antibody (51,064, 1:100, Proteintech). The IP targets were disassociated from the immobilized antibodies on AminoLink Plus Resin. Then, the eluted proteins were resolved using SDS–PAGE through 10% gels and subjected to by Western blot using mouse anti-PTP1B monoclonal antibody (201,974, 1:1000, Abcam) or rabbit anti-Src antibody (1:2000, Proteintech).

### 2.7 Western blotting analysis

Cultured cells and tumor tissues from nude mice were lysed with ice-cold RIPA buffer containing freshly added PMSF. Total protein was quantified using a BCA Protein Assay Kit (Beyotime, Guangzhou, China). Cell lysates  (40ug) were separated by SDS–PAGE and transferred onto a PVDF membrane (Millipore, Billerica, MA, USA). Membranes were blocked in 5% bovine serum albumin (BSA) in 1× Tris-buffered saline containing 0.05% Tween 20 (TBST) for 1 h at room temperature and then incubated with primary antibodies overnight at 4°C, followed by incubation with HRP-conjugated secondary antibodies for 1.5 h at room temperature. The primary antibodies used were as follows: anti-PTPN1 antibody (201,974, 1:1000, Abcam), anti-HA antibody (51,064, 1:2000, Proteintech), anti-Src antibody (19,096, 1:2000, Proteintech), and anti-β-actin antibody (60,008–1, Proteintech). The secondary antibodies used were as follows: anti-rabbit (7404, CST) and anti-mouse (7076, CST). The bands were visualized using enhanced chemiluminescence (ECL) reagent (WBULS0500, Millipore) with the Tanon 5200 system (Tanon, Shanghai, China). The Human Phospho-Kinase Array (ARY003C, R&D Systems) was used according to the array procedure, and the intensities were analyzed by Image J.

### 2.8 In vivo tumor xenograft study

Five-to seven-week-old female BALB/c nude mice were randomized and assigned to the control or experimental groups. A lung metastasis model was established by tail vein injections of 1 × 105 cells and was analyzed at 6 weeks after injection. Before the mice were sacrificed, we used PET-CT to evaluate lung metastasis in each group. 18-FDG was injected into the tail vein, and mice were scanned after 30 minutes. The SUV value was analyzed in each group, and then the mice were sacrificed. The lung metastases were detected by HE staining.

### 2.9 Statistical analyses

Statistical analyses were performed using GraphPad Prism software with unpaired t-test with Welch’s correction for comparison of two groups or one-way ANOVA with Tukey’s test for comparing more than two groups. In figures, unless otherwise noted, data are presented as the means ± SEM, where P ≤ 0.05 (*), P ≤ 0.01 (**), P ≤ 0.001 (***), and P ≤ 0.0001 (****) were considered statistically significant, and ‘NS’ indicates not statistically significant.

All experiments were performed at least three repetitions to ensure the accuracy of data.

## Results

PTP1B can promote tumor progression in breast cancer, colon cancer and prostate cancer. However, the clinical and biological functions of PTP1B in MM are unknown. In this study, we hypothesized that PTP1B is involved in the progression of MM. We detected the expression of PTP1B in human melanoma tissues and evaluated its function in melanoma metastasis in vivo and in vitro. Moreover, human phospho-kinase array and co-IP analysis revealed a critical role for PTP1B in melanoma metastasis through dephosphorylating Src.

PTP1B expression is highly elevated in malignant melanoma and correlates with poor survival rates

**Figure 1.**
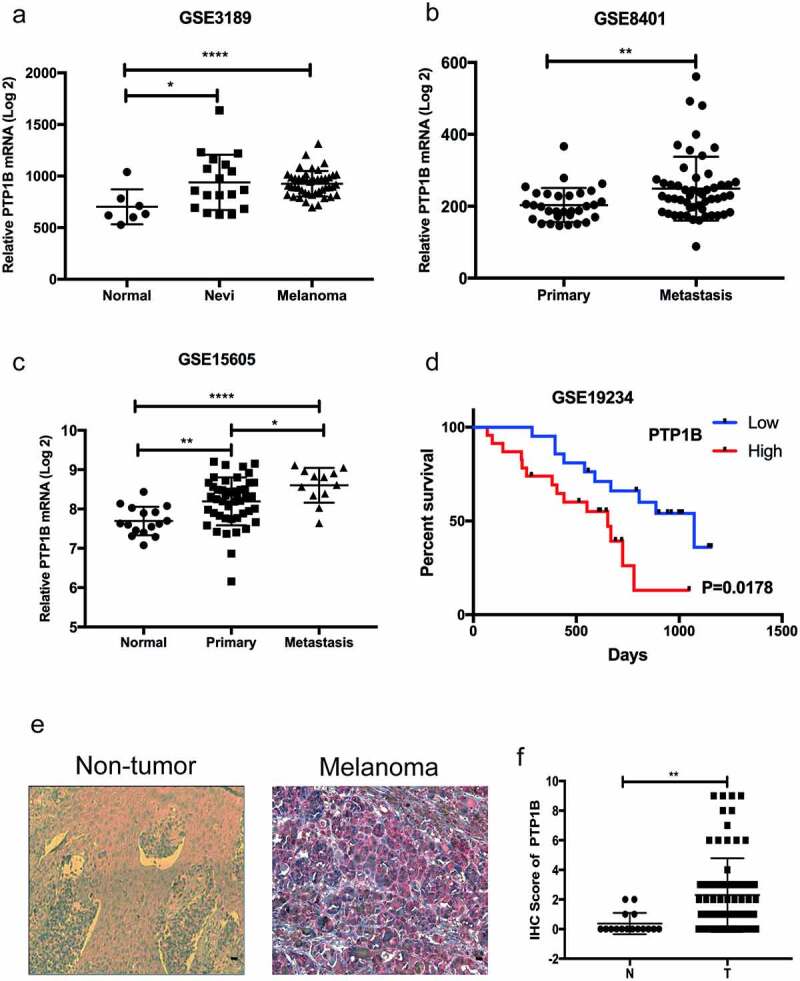
PTP1B expression is highly elevated in malignant melanoma and correlates with poor survival

PTP1B expression in melanoma and noncancerous tissues was analyzed in samples from GEO databases. As shown in Figure 1a, PTP1B mRNA was overexpressed in melanoma compared with normal tissues. Moreover, PTP1B expression was upregulated in metastatic melanoma compared with primary melanoma (Figure 1b&c). Survival analysis showed that PTP1B overexpression was related to poor survival rates in patients (Figure 1d). All these data indicate that PTP1B may promote melanoma progression. Therefore, we used AEC (Aminoethyl carbazole) IHC to detect the protein expression of PTP1B in melanoma and nontumor sample tissues. As shown in Figure 1e, PTP1B expression was detected in the cytoplasm (red color) and was upregulated in melanoma samples compared with nevi (Figure 1f). Collectively, these results indicate that PTP1B is a tumor promoter for MM.

A. PTP1B mRNA expression analysis in MM, nevi and normal tissues (GSE3189; Normal, n = 7; Nevi, n = 18; Melanoma, n = 45).B. PTP1B mRNA expression analysis in primary and metastatic tissues (GSE8401; Primary, n = 31; Metastasis, n = 52).C. PTP1B mRNA expression analysis in normal, primary and metastatic tissues (GSE15605; Normal, n = 16; Primary, n = 46; Metastasis, n = 12)D. Overall survival of MM patients based on PTP1B mRNA expression (GSE19234; High, n = 23; Low, n = 21).E. PTP1B protein expression in nontumor and melanoma tissues by immunohistochemical AEC staining. Original magnification 400 × .F. PTP1B expression was analyzed in nontumor and melanoma tissues (Normal, n = 18; Melanoma, n = 82).

## PTP1B deficiency inhibits the horizontal migration of melanoma cells

**Figure 2.**
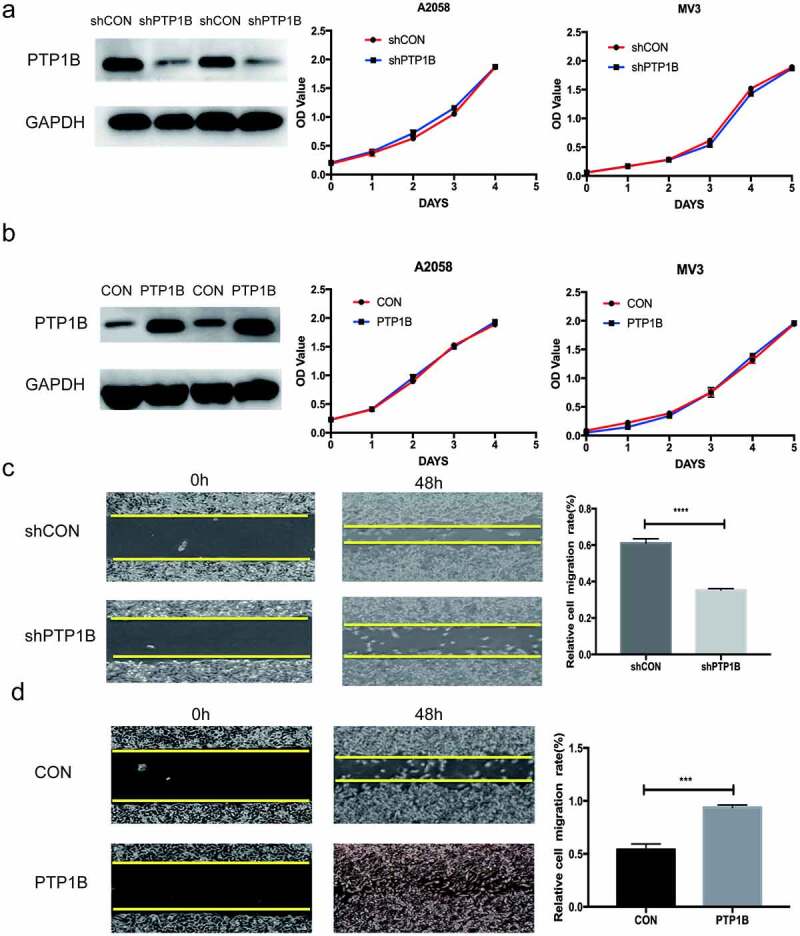
PTP1B deficiency inhibits the horizontal migration of melanoma cells without affecting cell proliferation

We used lentivirus to establish PTP1B-upregulated and PTP1B-downregulated A2058 and MV3 cells and detected proliferation in the experimental and control groups by performing a CCK-8 assay. As shown in Figure 2a&b, knockdown or overexpression of PTP1B had no effect on the growth of melanoma cells. Therefore, we used a wound healing assay to further analyze the biological role of PTP1B in horizontal cell migration. As shown in Figure 2c, downregulation of PTP1B significantly inhibited horizontal migration. In contrast, upregulation of PTP1B promoted horizontal migration of melanoma cells.

A The proliferation ability of A2058 and MV3 cells with knockdown of PTP1B was assessed by the CCK-8 assay (OD value 450 nm).B The proliferation ability of A2058 and MV3 cells with overexpression of PTP1B was assessed by the CCK-8 assay (OD value 450 nm).C The horizontal migration ability of A2058 cells with knockdown of PTP1B was assessed by the wound healing assay at 48 h.D The horizontal migration ability of A2058cells with overexpression of PTP1B was assessed by the wound healing assay at 48 h.

## PTP1B-overexpressing melanoma cells promote vertical migration and invasion

**Figure 3.**
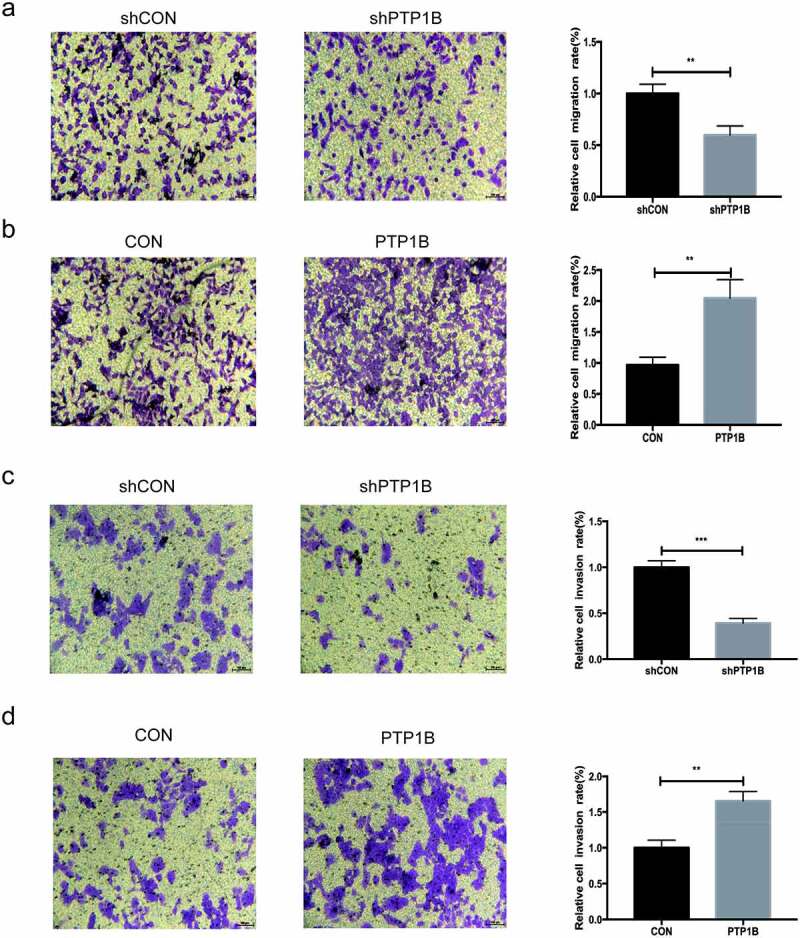
PTP1B-overexpressing melanoma cells show increased vertical migration and invasion

Similar to the wound healing assay, the vertical migration assay (Figure 3a&b) showed that downregulation of PTP1B significantly inhibited vertical cell migration and that upregulation of PTP1B promoted melanoma vertical cell migration. Then, melanoma cell invasion was evaluated (Figure 3c&d). Knockdown of PTP1B significantly inhibited cell invasion. In contrast, overexpression of PTP1B promoted melanoma cell invasion. Therefore, inhibition of PTP1B suppressed MM cell migration and invasion.

A&B The vertical migration ability of MV3 cells with knockdown or overexpression of PTP1B was assessed by the Transwell migration assay.C&D The invasion ability of A2058 cells with knockdown or overexpression of PTP1B was assessed by the Transwell invasion assay.

## PTP1B promotes tumor metastasis in vivo

**Figure 4.**
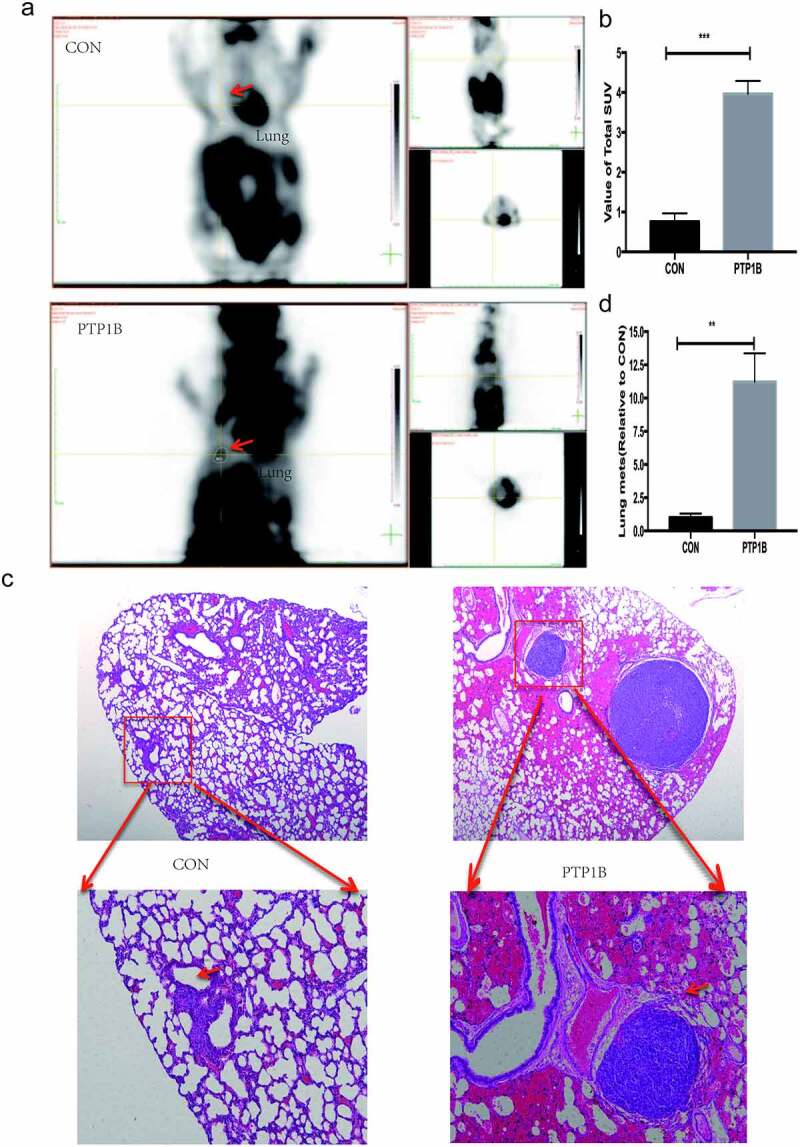
PTP1B promotes tumor metastasis in vivo

Lung is the commonest site for the occurrence of melanoma distant metastasis, and the prognosis of patients with lung metastasis is poor, median survival with lung metastasis was only 7 months. As PTP1B influence the metastasis of melanoma in vitro, we further confirm this function in vivo. A lung metastasis model was used to evaluate the effects of PTP1B in vivo. PET-CT images (Figure 4a) showed that the lung area (red arrow) was obviously more metabolically active in the OE group than in the control group. Moreover, the SUV value in the lung area was four times higher in the PTP1B OE group than in the control group (Figure 4b), which means that the OE group had more lung tumors than did the control group. HE staining was used to detect the number of metastases in the lung area. As shown in Figure 4c&d, the number and area of metastases in the OE group were ten times greater than those in the control group. These results again show that PTP1B could promote the metastasis of MM.

A. PET-CT images showing the lung metastasis area (red arrow).B. Analysis of the SUV value in the lung metastases.C. Images of lung metastases subjected to HE staining. Original magnification, 40× and 200 × .D. Analysis of the relative lung metastasis based on the number and area of metastases.

## PTP1B regulates melanoma cell migration by interacting with Src

**Figure 5.**
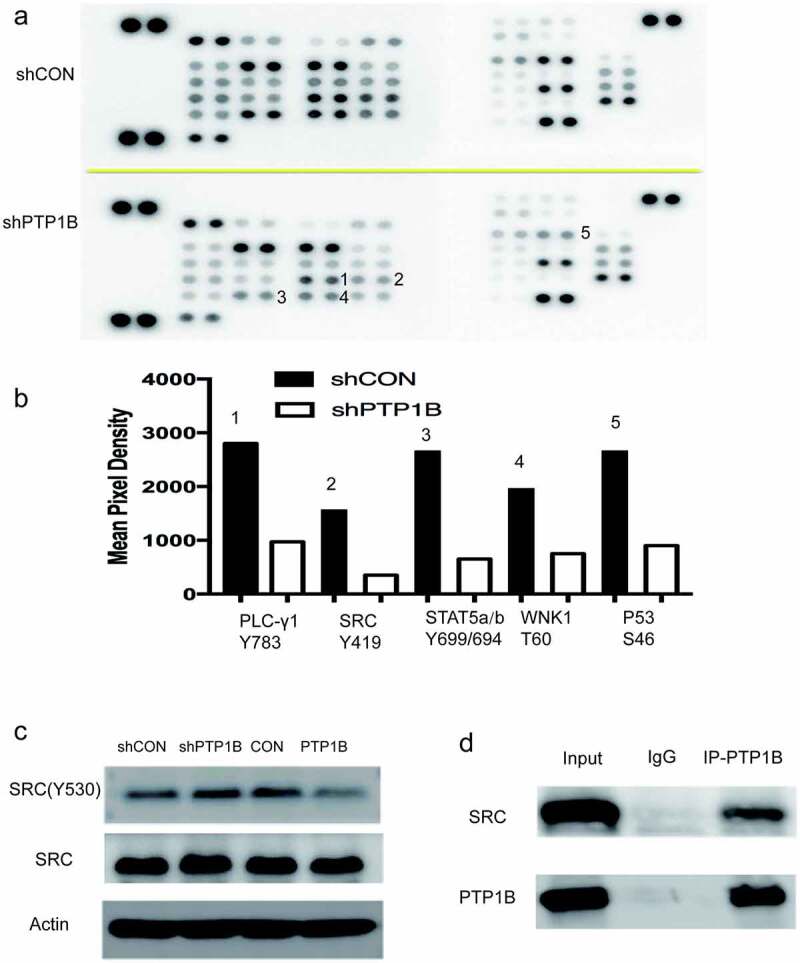
PTP1B regulates melanoma cell migration by interacting with Src

As PTP1B is a tyrosine phosphatase, the Human Phospho-Kinase Array was used to analyze the potential protein(s) affected by PTP1B. Figure 5a shows that the phosphorylation level of many proteins was inhibited in PTP1B-knockdown cells. As shown in Figure 5b, the phosphorylation of PLC, SRC, STAT5, WNK1 and P53 was decreased. PTP1B is reported to interact with Src and STAT5, so the phosphorylation of Src and STAT5 was detected by Western blots, and Src Tyr530 levels were measured in cells with up – and downregulated PTP1B expression. The phosphorylation level of Src at Tyr530 was increased in PTP1B-downregulated cells and decreased in PTP1B-overexpressing cells (Figure 5c). Co-IP assays showed that PTP1B could interact with Src (Figure 5d), which was previously reported in colon cancer. Together, these results show that PTP1B promotes MM metastasis and predicts poor survival rates.

A. Phosphorylation levels of proteins in A2058 cells with knockdown of PTP1B were detected in the Human Phospho-Kinase Array.B. Mean pixel density shows changes in the phosphorylation status of proteins.C. The phosphorylation level of Src Tyr530 was detected in cells with knockdown or overexpression of PTP1B.D. Proteins pulled down with PTP1B-HA antibody were evaluated by western blotting for the presence of PTP1B and Src.

## Discussion

In this study, we report that PTP1B is overexpressed in MM tissues and that downregulation of PTP1B suppresses the migration and invasion of melanoma cells. Moreover, we discovered an association between PTP1B and Src in the progression of MM. As a tyrosine phosphatase, PTP1B was reported to be involved in the progression of many cancers by dephosphorylating key proteins. In esophageal squamous cell carcinoma, PTP1B promotes cell invasion and migration by dephosphorylating MYH9 at Y1408, which results in increased EGFR expression [27]. In colorectal carcinoma, PTP1B directly dephosphorylates PITX1 at Y160, Y175 and Y179, which destabilizes PITX1 and consequently results in downregulation of the PITX1/p120RasGAP axis [28]. However, the biological function of PTP1B in MM is unknown.

The carboxyl terminal residue Tyr530 in human c-Src is an important site for the regulation of c-Src tyrosine kinase activity [29,30]. When Tyr530 is phosphorylated, it binds to the SH2 domain of Src. The combination of the two makes the molecule curl, and then the catalytic pocket is covered, which will inhibit the tyrosine kinase activity of c-Src. Protein tyrosine phosphatase family members were found to be physiological regulators of Src [31–33]. Experiments show that PKC can act on PTPα and that the catalytic activity of PKC increases and enhances PTPα activity, which results in the automatic phosphorylation of Tyr419 to further activate Src [34]. Another protein tyrosine phosphatase, PTPλ, can dephosphorylate c-Src. This dephosphorylation can occur at the Tyr419 site but mainly occurs at the Tyr530 site [35]. Moreover, PTP1C can dephosphorylate the Tyr419 and Tyr530 sites but has a stronger effect on the Tyr530 site [36]. Using the Human Phospho-Kinase Array, we found that the phosphorylation level of Tyr419 was inhibited when PTP1B was downregulated. Because Src activity is mainly reduced after phosphorylation of the Tyr530 site, we detected the phosphorylation level at Tyr530. As shown in Figure 5c, the phosphorylation level at Tyr530 was inhibited in the PTP1B-overexpressing group, which indicated that PTP1B might dephosphorylate Src at Tyr530. Moreover, a co-IP assay showed that PTP1B could interact with Src. All these findings indicate that PTP1B might promote the metastasis of MM by interacting with Src and dephosphorylating the Tyr530 site. However, further studies are required to further elucidate the detailed mechanism between PTP1B and Src.

In summary, PTP1B is overexpressed in MM tissues and promotes metastasis by binding Src and dephosphorylating the Tyr530 site. Therefore, elucidating the function of PTP1B could pave the way for improved therapy for MM.

## Conclusion

The present study provides new insights into PTP1B function in human melanoma tissues. Our data showed that downregulation of PTP1B expression suppresses and overexpression of PTP1B promotes cell migration and invasion. Moreover, overexpression of PTP1B causes significant progression of established lung metastasis in nude mice by targeting Src, suggesting that PTP1B is as a novel therapeutic target for the management of MM.

## Abbreviation

MM (Malignant Melanoma), PTP1B (Protein tyrosine phosphatase 1B), PTPN1 (Protein Tyrosine Phosphatase Non-Receptor Type 1), PET-CT (positron emission tomography / computedtomography), SUV (standard uptake value), AEC (Aminoethyl carbazole), IHC Immunohistochemistry, GEO (Gene Expression Omnibus).
